# Effect of Smartphone-Based Messaging on Interns and Nurses at an Academic Medical Center: Observational Study

**DOI:** 10.2196/66859

**Published:** 2025-04-17

**Authors:** Sankirth Madabhushi, Andrew M Nguyen, Katie Hsia, Sucharita Kher, William Harvey, Jennifer Murzycki, Daniel Chandler, Michael Davis

**Affiliations:** 1 Department of Medicine, UMass Chan Medical School, 55 N Lake Ave, Worcester, MA, 01655, United States, 1 (774) 442-2173; 2 Tufts University School of Medicine, Boston, MA, United States; 3 Department of Medicine, Tufts Medical Center, Boston, MA, United States; 4 Information Systems, Tufts Medical Center, Boston, MA, United States; 5 Department of Pediatrics, Tufts University School of Medicine, Boston, MA, United States

**Keywords:** secure messaging, alert fatigue, burnout, clinical communication, TigerConnect, healthcare communication, text, texting, messaging, app, application, smartphone, digital health, digital technology, digital intervention, medical informatics, messaging patterns

## Abstract

**Background:**

Digital communication between nurses and medicine interns plays a crucial role in patient care. However, excessive messaging may contribute to alert fatigue, potentially affecting workflow efficiency and clinical decision-making. Although prior research has examined general messaging behaviors among clinicians, few studies have specifically analyzed messaging patterns between nurses and interns, who serve as primary points of contact in inpatient care.

**Objectives:**

This study aims to quantitatively characterize messaging patterns between the primary nurse and primary provider (ie, medicine intern) of hospitalized patients at an academic medical center in order to identify communication burdens and potential inefficiencies. By identifying trends in message volume, timing, and response rates, we seek to inform strategies to optimize communication workflows and mitigate alert fatigue.

**Methods:**

At a large academic hospital (Tufts Medical Center, Boston, MA), we analyzed secure messaging transactions between internal medicine interns and nurses across three medical-surgical units over 6 months. Transaction metadata, time stamps, and unique message tokens were extracted. Data processing was performed using Python, Microsoft Excel, and R. Message volume, interaction frequencies, and response times were analyzed using measures of central tendency and statistical tests of significance.

**Results:**

A total of 61,057 unique messages were exchanged between interns and nurses, with interns exchanging 2.5 times more messages per day with nurses than vice versa (*P*<.001). Messaging volume exhibited diurnal variation, indicating periods of increased communication burden. Interns read messages from nurses within a median of 35 (range: 0‐3589) seconds, whereas nurses read messages from interns within a median of 26 (range: 0‐3584) seconds (*P*<.001). The longest message response delays occurred at 4 AM, whereas the shortest occurred at 8 AM.

**Conclusions:**

Interns experience a significantly higher messaging burden than nurses, with distinct peaks in message volume during morning rounds and overnight shifts. These findings suggest a need for interventions such as optimized digital communication protocols to reduce nonessential messaging and alert fatigue. Future research should explore the effectiveness of these interventions in enhancing workflow efficiency and the development of both in-person and digital interventions to optimize communication workflows and mitigate alert fatigue.

## Introduction

As health care organizations implement secure messaging software to modernize communication among clinicians and address the shortcomings of pagers, a growing body of literature has described patterns in secure messaging transactions [1,2]. Small et al [3,4] describe an uneven burden of secure messaging on medicine providers, while qualitative analysis reveals clinicians’ concerns with alert fatigue from digital communication. Alert fatigue is a phenomenon in health care settings where providers experience a high frequency of clinical notifications, leading to desensitization of safety alerts, increased distractions, missed alerts, or cognitive overload. Such desensitization can significantly contribute to clinician burnout and affect patient care [5,6]. To our knowledge, few studies have examined the specific communication patterns between nurses and medicine interns on secure messaging platforms. Existing research provides general insights into messaging behaviors but lacks in-depth analysis of how messaging patterns between these specific groups—who often serve as the first point of contact in inpatient care—differ in terms of volume or timing [7,8].

Our institution (Tufts Medical Center, Boston, MA) implemented TigerConnect, a clinical communication and collaboration platform, in April 2022, and in turn, disabled the secure chat feature in the electronic medical record “Epic.” Although TigerConnect has yielded efficiencies to clinical workflows, such as consult requests, operating room throughput, closure of radiology alerts, and emergency responses, alert fatigue from messages was cited as a reason for decreased job satisfaction on end-of-year house staff surveys. This study aims to characterize the messaging patterns between nurses and medicine interns to inform interventions to mitigate alert fatigue among two groups of clinicians with exceptionally high rates of burnout [9].

## Methods

### Study Setting and Data Collection

Our study was conducted at Tufts Medical Center, a 415-bed tertiary care academic institution in Boston, MA. We focused on TigerConnect transactions between 25 internal medicine interns and 96 nurses from three medical-surgical inpatient units, excluding the intensive care unit and emergency department, across 5 months from June 2022 through December 2022. We also collected transaction metadata, time stamps, and unique message tokens.

### Data Analysis

We compiled the data using Python and Microsoft Excel. A dataset of messages exchanged between the interns and nurses was cleaned and analyzed using R, specifically using the *dpylr* package. We defined an “active” intern or nurse as someone who sent or received a message during a given period. Interaction frequencies were summarized using measures of central tendency. The time lag between message delivery and reading was calculated by subtracting the delivery time stamp from the read time stamp, with messages read over an hour later excluded to avoid skewing caused by outliers. Average interactions were compared using a 2-sided Welch *t*-test. This test was chosen due to unequal variances between the two groups. Median comparisons of time delays were compared using the Mann-Whitney test with an α of .05.

### Ethical Considerations

The Tufts Health Sciences Campus institutional review board reviewed this study and determined that it did not constitute human subjects research; thus, it was exempt from institutional review board approval. Data from this study presents only metadata from message transactions between 25 interns and 96 nurses, including time stamps, delivery confirmations, and read receipts. To protect privacy, individual interns and nurses were deidentified, and transactions were aggregated, as the analysis focused on overall trends rather than individual messaging behavior. Individual interns and nurses were deidentified by replacing the names of individual interns with an “intern” label and replacing the names of individual nurses with a “nurse” label. Furthermore, the actual content of any messages was not accessed, in accordance with institutional policy and the scope of this study.

## Results

### Message Volume and Patterns

**Figure 1.**
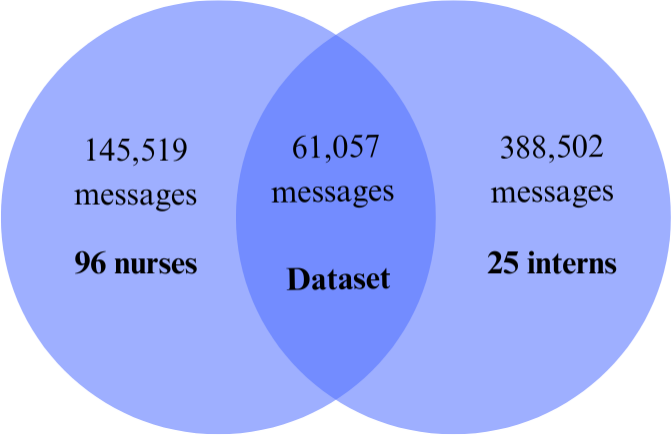
Message transactions between 96 nurses and 25 interns over a 5-month period, showing the total number of messages exchanged with nurses, with interns, and specifically between nurses and interns.

**Figure 2.**
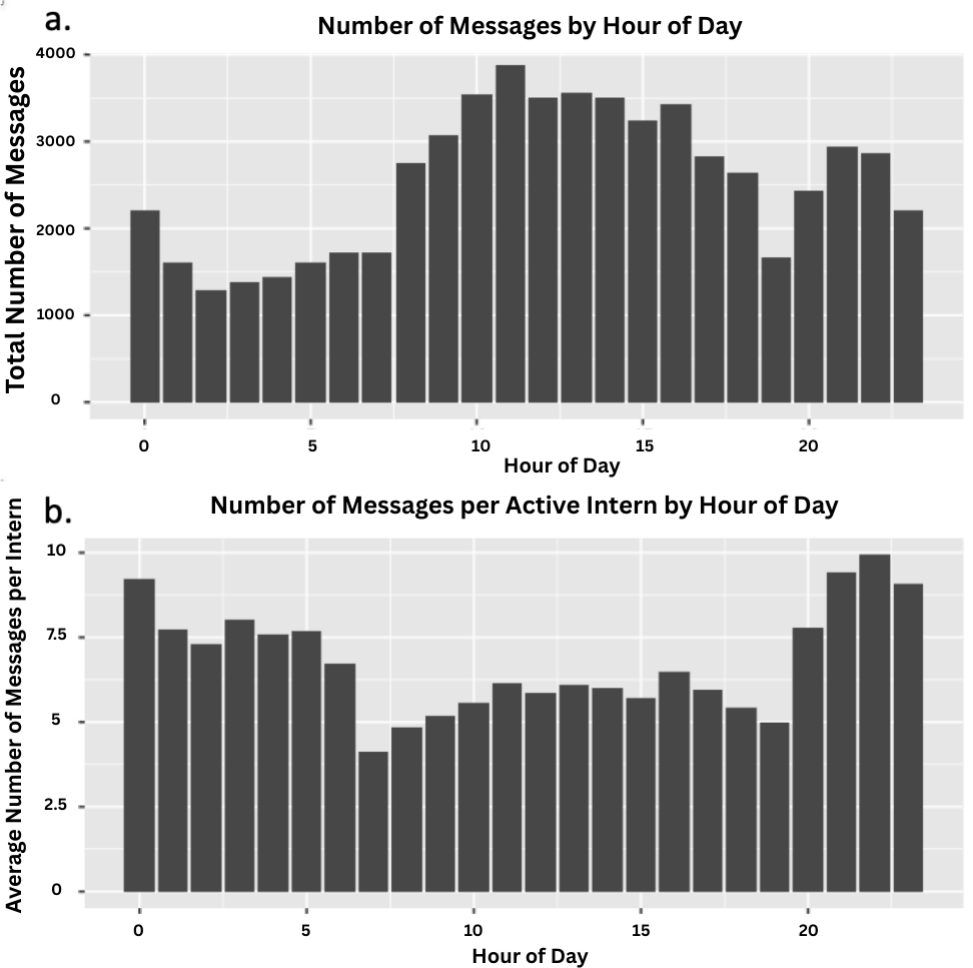
(A) Hourly distribution of total message volumes exchanged between nurses and interns by hour of the day. (B) Hourly distribution of message volumes per active intern by hour of the day.

A total of 145,519 (sent and received) nurse messages and 388,502 intern messages were collected over the study period, with 61,057 unique messages exchanged between interns and nurses (Figure 1). An active intern exchanged on average 39.8 messages (range: 26.8‐50.6) with nurses per day, while an active nurse exchanged on average 15.3 messages (range: 2.3‐37.2) with interns per day (*P<*.001). Each intern covers up to 9 patients, whereas each nurse covers typically up to 4 patients. On average, the total number of messages exchanged over a day peaked at 11 AM. (Figure 2A). Typically, 13 interns work from 7AM to 6PM and 2 interns work from 6PM to 7AM. On average, the number of messages per active intern peaked at 10 PM. (Figure 2B).

### Message Response Times

On average, interns read messages from nurses within a median of 35 seconds (range: 0‐3589), whereas nurses read messages from interns within a median of 26 seconds (range: 0‐3584) (*P*<.001). The daily nadir and peak of delivered-to-read delays occurred at 4 AM and 8 AM, respectively.

## Discussion

### Principal Results

Similar to Small et al [3], we found a significantly disproportionate message burden between interns and nurses. On average, interns exchanged 2.5 times more messages per day with nurses than nurses exchanged with interns (Figure 1). This difference is reasonable, as interns care for twice as many patients as nurses. Our study also highlights diurnal variation in message volume (Figure 2). The highest volume of messages occurred mid-morning, when interns would participate in rounds and nurses would administer morning medications. During this period, critical questions and information needs to be communicated, and the inability to communicate in person likely led to increased message volume. The overnight peak in messages per active intern was likely driven by the night-time interns’ lack of proximity to nurses, making in-person communication more difficult, as well as the reduced number of interns on duty at night (2 night-time interns vs 13 day-time interns).

The mid-morning peak of total messages suggests the need for interventions to reduce nonessential messages during specified time windows—for example, “quiet hours” sponsored by hospital leadership. Previous studies have demonstrated that cooperative programs focusing on decreasing nonurgent pages can lead to a significant reduction in their number and increase uninterrupted time at night and during educational periods, thereby improving residents’ work environment [10,11]. However, success depends on adherence and the ability to balance minimizing disruptions with ensuring timely responses to critical issues [10,11]. The overnight peak in messages suggests the need for investigating the effect of more proactive in-person check-ins between interns and nurses. While structured face-to-face communication—particularly during rounds—enhances team cohesion, reduces cognitive load, and fosters psychological safety, its impact outside of rounds remains sparsely investigated [12,13].

Future studies should involve implementing and evaluating the long-term effectiveness of such interventions across diverse hospital settings with different communication avenues (secure messaging, vs paging, etc), exploring how they influence provider well-being and workflow efficiency. Additionally, research should examine strategies to optimize these interventions, such as integrating quiet hours with structured escalation protocols, and assessing the feasibility of brief but frequent in-person check-ins outside of rounds.

Due to institutional policies restricting access to message content, a breakdown of message types (eg, consults, diet change requests) could not be analyzed. However, by categorizing messages based on content, one could use natural language processing to help identify specific communication patterns that contribute to alert fatigue and guide specific interventions, such as streamlining nonurgent alerts or improving response workflows. Future studies should explore the possibility of using deidentified message content to develop a more nuanced understanding of secure messaging dynamics.

While this study was conducted at a single institution—Tufts Medical Center, the messaging patterns observed between interns and nurses are likely reflective of broader trends in academic medical centers with similar communication infrastructures and staffing structures. Many academic hospitals use secure messaging platforms like TigerConnect and face similar challenges related to alert fatigue, communication breakdowns, and clinician burnout. Thus, the findings from this study can offer valuable insights for other institutions seeking to optimize clinical communication, reduce unnecessary messaging, and implement interventions that support clinician well-being.

### Limitations

The study is limited by its observational nature and setting in a single institution, which may limit the generalizability of its findings. Causal conclusions could not be drawn because patterns were only observed without testing for direct effects. Other factors, such as workload, staffing levels, and communication protocols, were not analyzed and could have influenced message patterns. Future studies should include these factors to better understand messaging behavior. Institutional policies restricting message access prevented analysis of message types (eg, consults, diet change requests).

### Conclusions

This study quantitatively characterizes messaging patterns between nurses and medicine interns at a large academic hospital in Boston, MA, revealing an imbalance with interns exchanging significantly more secure messages. Although secure messaging systems like TigerConnect offer practical and important functions to clinicians, these findings highlight the potential for message overload. Future work should focus on investigating proactive communication strategies to optimize its use, reducing burden and improving job satisfaction while maintaining the benefits of digital communication for patient care.
